# Targeting membrane proteins for antibody discovery using phage display

**DOI:** 10.1038/srep26240

**Published:** 2016-05-18

**Authors:** Martina L. Jones, Mohamed A. Alfaleh, Sumukh Kumble, Shuo Zhang, Geoffrey W. Osborne, Michael Yeh, Neetika Arora, Jeff Jia Cheng Hou, Christopher B. Howard, David Y. Chin, Stephen M. Mahler

**Affiliations:** 1 Australian Institute for Bioengineering and Nanotechnology, The University of Queensland, St Lucia Queensland 4072 Australia; 2 Faculty of Pharmacy; King Abdulaziz University, 21589 Jeddah, Saudi Arabia; 3 Queensland Brain Institute, The University of Queensland, St Lucia Queensland 4072 Australia

## Abstract

A critical factor in the successful isolation of new antibodies by phage display is the presentation of a correctly folded antigen. While this is relatively simple for soluble proteins which can be purified and immobilized onto a plastic surface, membrane proteins offer significant challenges for antibody discovery. Whole cell panning allows presentation of the membrane protein in its native conformation, but is complicated by a low target antigen density, high background of irrelevant antigens and non-specific binding of phage particles to cell surfaces. The method described here uses transient transfection of alternating host cell lines and stringent washing steps to address each of these limitations. The successful isolation of antibodies from a naive scFv library is described for three membrane bound proteins; human CD83, canine CD117 and bat CD11b.

Membrane proteins are extremely attractive as targets for research, diagnostic and therapeutic applications. Specific membrane proteins can define certain cell types or stages of development, in particular subsets of immune cells are defined by the presence or absence of various Cluster of Differentiation (CD) markers^1^. Additionally, cells in a diseased state have altered normal levels of membrane receptor proteins; for example, the expression of the receptor Her2 is up-regulated in over 20% of breast cancers^2^. As such, antibodies against membrane proteins are highly sought after both as research reagents and for therapeutic purposes. There are nearly 50 monoclonal antibodies (mAbs) approved or under review as therapeutic drugs throughout Europe and the United States, and of these 61% target proteins that are present on the cell surface (http://www.antibodysociety.org/news/approved_mabs.php, updated May 26, 2015).

New mAbs may be isolated by several different methods. Animal immunisation in conjunction with hybridoma technology^3^ remains the most common method, especially for mAbs used as laboratory reagents, as it is a robust and licence-free method which produces mAbs of high affinity. However, mouse-derived mAbs have limited use in the clinic, as they are immunogenic in a human host^4^. These mAbs require protein engineering strategies to create chimeric or humanized mAbs to reduce their immunogenicity in a human patient^5^.

To avoid mouse-derived sequence entirely, mAbs may also be isolated using transgenic, humanized mice^6^ (followed by hybridoma technology), or using antibody library display technologies^7^. The former technology is very expensive and hindered by intellectual property protection, while display technologies are relatively cheap and available to research laboratories. Phage display is the most common of the display technologies (which also include ribosome display, yeast display and mammalian display). It was first described by George P. Smith, who demonstrated that proteins or peptides could be displayed on the surface of filamentous phage via a genetic fusion with a phage coat protein in the phage genome^8^. The technology was later extended to allow cloning into simpler phagemid vectors and the creation of libraries of antibody fragments (scFv or Fab), cloned from human blood and displayed on phage^9^,^10^. These libraries were then screened against immobilized target proteins to isolate antibodies of defined specificity, in a process known as “biopanning”^11^.

Methodology for biopanning antibody libraries on soluble, purified proteins is well established^12^,^13^, however the successful isolation of mAbs using phage display is highly dependent on the quality of the antigen being used. The antigen needs to be presented to the phage library in as close to its native conformation as possible. This can be difficult to achieve for membrane proteins, which contain hydrophobic transmembrane domains, and may have extended extracellular regions made up of multiple domains, or furthermore may be part of a multi-subunit cluster of proteins. A solution to this problem is to biopan libraries using whole cells as the antigen source, thereby maintaining a native conformation of the protein.

Whole cell biopanning however, has many difficulties. Firstly, there exists a high background of non-relevant proteins, and secondly, the target protein may be in low abundance in comparison to this high background. Thirdly, phage particles have the propensity to non-specifically adsorb to cell surfaces via coat proteins not associated with the antibody fragment and these phage will be eluted alongside specifically bound phage.

We have optimized a method for biopanning antibody phage libraries on whole cells that addresses each of these problems. The method utilises transient transfection of the target protein along with Green Fluorescent Protein (GFP), to both increase the target protein density and provide a means to select for cells with high level expression of cell surface protein using Fluorescence Activated Cell Sorting (FACS). The host cell line is alternated between Chinese Hamster Ovary (CHO) cells and Human Embryonic Kidney (HEK) cells with each round of biopanning to help eliminate background binders. A low pH wash is incorporated to remove phage which are present through non-antibody binding.

The method was initially developed using human CD83 as a model system. The CD83 antigen is expressed on activated dendritic cells^14^, and we have previously expressed the extracellular domain of CD83 and isolated antibodies by biopanning on the soluble protein^15^. We have now used transiently-expressed membrane-bound CD83 for optimisation of the cell-based biopanning method, using the soluble protein as a means of monitoring the success of the technique. We then optimized and tested the method using canine CD117 (c-Kit) and bat CD11b, for which no recombinant soluble material and no antibodies against the extracellular domains were available.

## Results

### Expression of membrane-bound antigens

**Figure 1:**
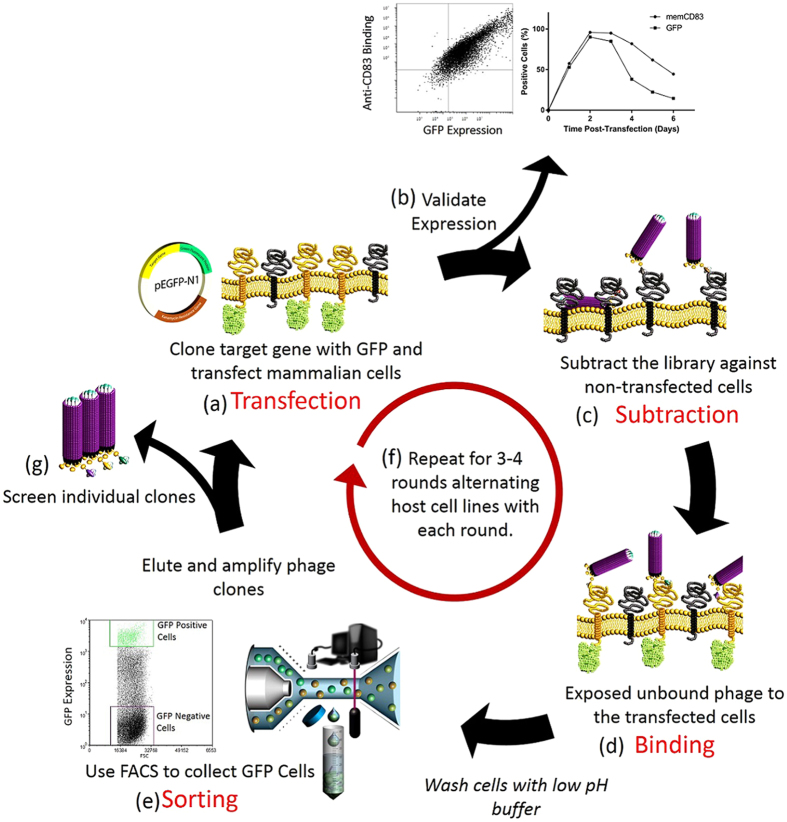
Overview of the methodology used for cell-based panning of membrane proteins. (**a**) The target membrane gene is cloned into pEGFP-N1 in-frame with GFP. Transfection into CHO or HEK cells results in expression of the membrane protein on the cell surface, with attached intracellular GFP, which gives (**b**) proportionality between GFP and target expression (shown here by flow cytometry analysis two days post-transfection of CD83-GFP transfected cells binding to anti-CD83 antibody). Expression of the target protein is maximal on Day Two post-transfection (shown here as a time-course flow cytometry assay showing the percentage of cells expressing CD83 and GFP up to 6 days post-transfection). (**c**) On Day Two post-transfection, the phage library is depleted against non-transfected cells, and then (**d**) the unbound phage is incubated with the transfected cells. (**e**) After washing with a low pH buffer, FACS is used to collect high GFP expressing cells, and the phage is eluted from the high-GFP cells and infected into *E. coli*. (**f**) The phage is amplified for the next round of panning, where the host transfection cell line in alternated between CHO and HEK cells for 3 to 4 rounds. (**g**) Individual clones are screened by flow cytometry after 3 to 4 rounds of panning.

Each of the target membrane proteins, including their trans-membrane domains, were cloned in-frame with GFP, such that the target protein is displayed on the extracellular surface of transfected cells and the GFP is intracellular, as a transmembrane fusion protein (Fig. 1a). To validate the surface expression, CHO cells transfected with CD83-GFP were probed with an antibody (3C12) to the extracellular domain of CD83^15^. Figure 1b shows that 3C12 binding was proportional to the GFP expression within the cells, confirming that CD83 was being expressed on the surface of the cells, and a time-course analysis of the transfected cells showed maximum detection of both CD83 and GFP was achieved on Day 2. Detection of GFP expression decreased at a higher rate than CD83 after Day 3, presumably due to the lower conformational stability of the GFP domain of the fusion protein, leading to decreased fluorescence.

### Cell-Based Biopanning Optimisation on CD83

Cells collected two days post-transfection were incubated for 1 hr with the human naive antibody phage library, after an initial depletion step against non-transfected cells (Fig. 1c,d). The cell-phage mixture was washed with a pH 5 buffer to remove non-specifically adsorbed phage and then sorted using FACS to collect only the cells expressing high levels of GFP (Fig. 1e). Bound phage were eluted from these cells using a pH 3 buffer, infected into *E. coli* and amplified for subsequent rounds of biopanning.

**Figure 2:**
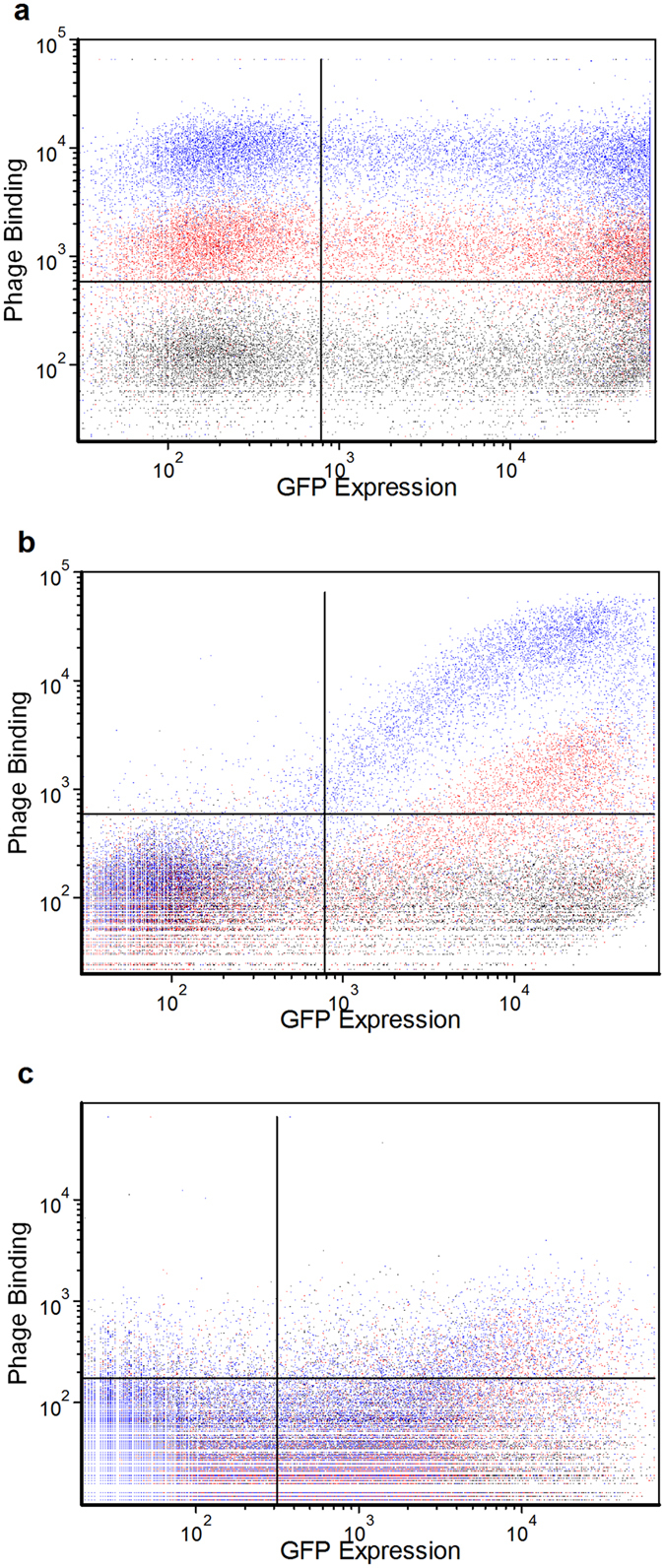
Flow cytometry analysis of phage pools after three rounds of biopanning. (**a**) Phage pools from biopanning CHO cells transfected with CD83-GFP. (**b**) Phage pools from biopanning alternating CHO and HEK cells, transfected with CD117-GFP. (**c**) Phage pools from biopanning alternating CHO and HEK cells, transfected with CD11b-GFP. Round 1 analysis is indicated in black, round 2 in red and round 3 in blue.

**Figure 3:**
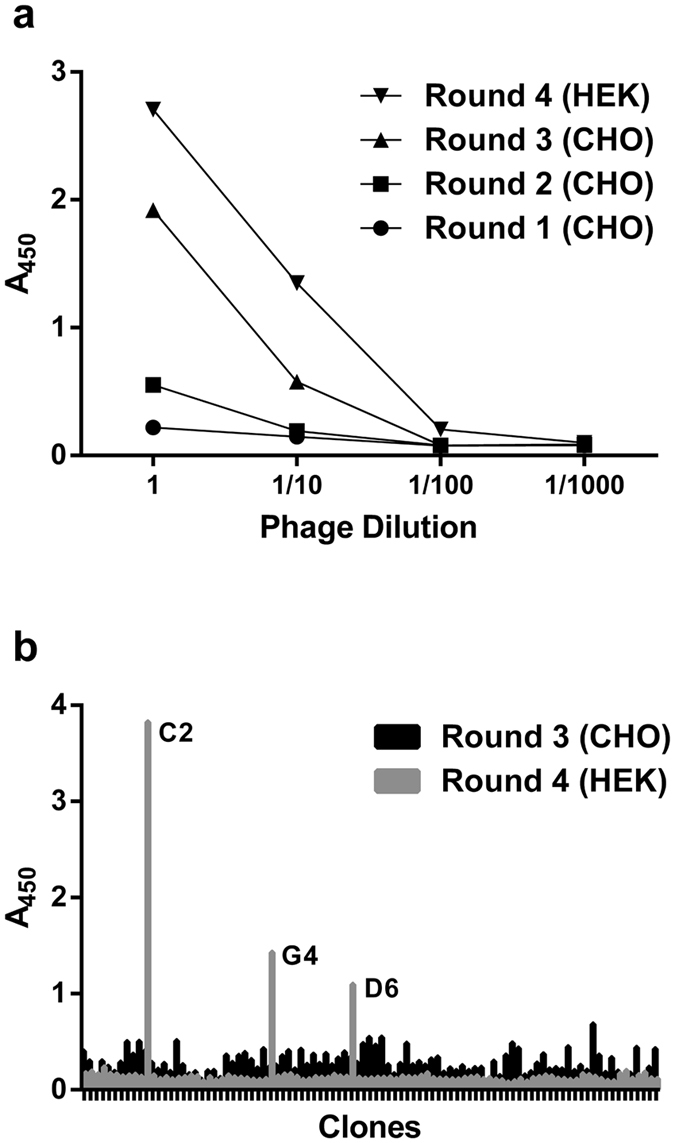
ELISA analysis of phage panned against membrane bound CD83-GFP, for binding to immobilized, soluble CD83 with detection using HRP-conjugated anti-M13 antibody. (**a**) Polyclonal analysis of dilutions of the phage pool from each round of biopanning. (**b**) Monoclonal analysis of rescued phage from randomly selected clones from Rounds 3 and 4 biopanning.

For the CD83 biopanning, three rounds were performed initially on transfected CHO cells. Flow cytometry was used to test the phage pools from each round of biopanning for binding to transfected cells. While the phage pools showed increased overall binding to the cells with each round of biopanning, there was no evidence of specificity towards the GFP expressing cells (Fig. 2a). However, when the pools were analysed using ELISA for binding to immobilized, soluble CD83 extracellular domain, it was evident that the third round pool did actually contain binders to CD83 (Fig. 3a). Thus, it was concluded that the number of CD83 binders within the pool, although present, was minimal compared to the number of binders against other cell surface antigens. Based on this outcome, a fourth round of biopanning was initiated against CD83-GFP transfected HEK cells, in order to eliminate the CHO cell binders. The ELISA result showed a further increase in binding of the phage pool to soluble CD83 after this fourth round (Fig. 3a). The flow cytometry analysis showed a lower overall binding to cells, but still no specificity towards GFP expressing cells indicating that the CD83 binders are still a minor population within the pool (data not shown).

With the knowledge that CD83 binders were present in the Rounds 3 and 4 phage pools, an analysis of 90 individual phage clones from each pool was performed using ELISA (Fig. 3b). No CD83-binding clones were identified from Round 3, indicating a frequency of less than 1 binder in 90 clones. In Round 4, three CD83-binding clones were identified; still a low frequency of 1 in 30 clones explaining the failure to distinguish these binders from the background binders using flow cytometry on the phage pools.

**Figure 4:**
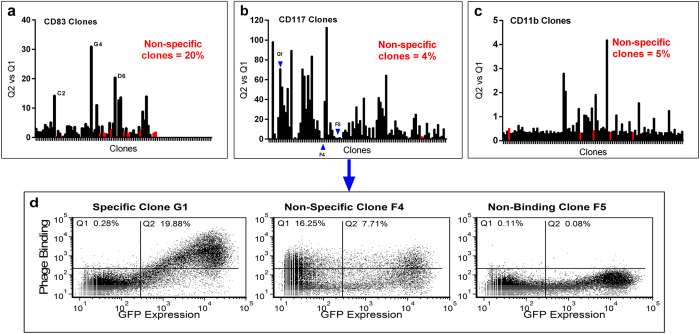
Monoclonal flow analysis of clones randomly selected from the final round of cell-based biopanning against each of the membrane bound antigens, for binding to CHO cells transfected with the respective GFP-tagged antigen. Clones were analyzed by the ratio of the percentage of cells showing both GFP expression and phage binding (Q2 quadrant) to the percentage of cells showing phage binding to non-GFP expressing cells (Q1 quadrant). (**a**) Phage clones from CD83-GFP biopanning; (**b**) Phage clones from CD117-GFP biopanning; (**c**) Phage clones from CD11b-GFP biopanning. Non-specific clones binding to an unknown irrelevant antigen on the CHO cell surface are highlighted in red. (**d**) Scattergrams showing example clones taken from the CD117-GFP panning that show specific binding (Specific Clone G1), non-specific binding (Non-Specific Clone F4) and no-binding (Non-Binding Clone F5).

Since soluble antigen is not always available for screening purposes, it was essential to show that positive clones could be detected by a method not requiring soluble protein, so the same clones from Round 4 were screened by flow cytometry on transfected CHO cells (Fig. 4a). The ratio of phage-bound, GFP expressing cells to phage-bound, non-GFP expressing cells was used to determine specificity of each clone. 20% of the clones were considered non-specific, binding to both GFP-expressing and non-GFP-expressing cells, explaining why no enrichment was detectable in the phage pools. However, nine clones showing specificity to GFP-positive cells were detected (Fig. 4a), including the same three clones (C2, G4 and D6) identified by soluble CD83 ELISA (Fig. 3b). These three clones were determined to be identical after sequencing the variable regions within the phagemid vector. The other six positive clones, which were not ELISA positive, were represented by four additional unique sequences. These clones may bind to an epitope that is displayed on the cell surface, but is not accessible when the protein is immobilized on plastic for ELISA. This result exemplifies the problems that occur when proteins change conformation upon immobilization. We previously panned the same library against immobilized CD83, isolating seven unique binders (unpublished data), but only one of these binders, 3C12, was able to bind native, cell-surface CD83.

### Cell-Based Biopanning Validation on CD117 and CD11b

In an attempt to improve the specificity and phage pool enrichment during the canine CD117 and bat CD11b biopanning campaigns, the transfection host cell line was alternated between each round of biopanning. There was no soluble canine CD117 or bat CD11b available for ELISA screening, therefore screening was performed by flow cytometry only. Alternating the host cell line greatly improved specificity by eliminating binders to host cell proteins, as indicated by the analysis of phage pools from each round of biopanning (Fig. 2b,c). Phage antibodies binding specifically to GFP-expressing cells were evident in the phage pools from Round 2, with increased binding in Round 3. The phage pools showed very little binding to non-GFP expressing cells, due to the change in host cell antigens between each round of biopanning. This is further evidenced in the monoclonal flow analysis for CD117 (Fig. 4b) and CD11b (Fig. 4c), where non-specific binders were reduced to 4–5% of the population, compared with 20% for the CD83 biopanning without alternating cell lines (Fig. 4a). Positive clones were identified by the ratio of their binding to GFP expressing cells to their binding to non-GFP expressing cells. The dynamic range of this ratio varies between the antigens, as it depends on the level of expression of each antigen. CD11b had one log lower GFP fluorescence than CD117, making the monoclonal screen less sensitive, but positive clones were still detectable.

**Figure 5:**
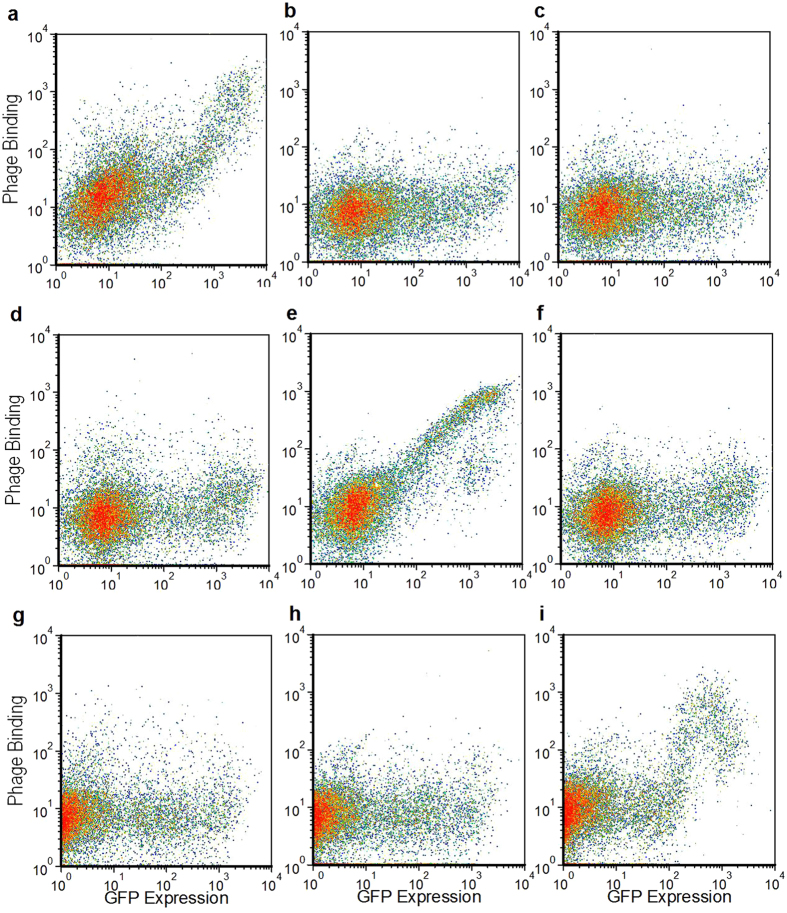
Flow cytometry test for specificity of clones isolated by cell-based biopanning on GFP-fused target antigens. CHO cells were transfected with CD83-GFP (**a**–**c**), CD117-GFP (**d**–**f**) or CD11b-GFP (**g**–**i**) and then probed with either Clone C2 isolated by biopanning of CD83-GFP (**a**,**d**,**g**), Clone A3 isolated by biopanning on CD117-GFP (**b**,**e**,**h**) or Clone H7 isolated by biopanning on CD11b-GFP (**c**,**f**,**i**).

As the CD83, CD117 and CD11b were transfected as GFP-fusion proteins, it was conceivable that the isolated phage clones were binding to either GFP (despite being expressed intracellularly) or another artefact of transfection. Therefore, the positive clones from each biopanning campaign were analysed for cross-reactivity against all of the transfected cell lines. All unique clones were analysed, and each showed phage binding specific for the transfected protein used for biopanning, giving confidence that the phage were binding to the cell-surface exposed region of their respective target antigens (representative clones shown in Fig. 5).

**Figure 6:**
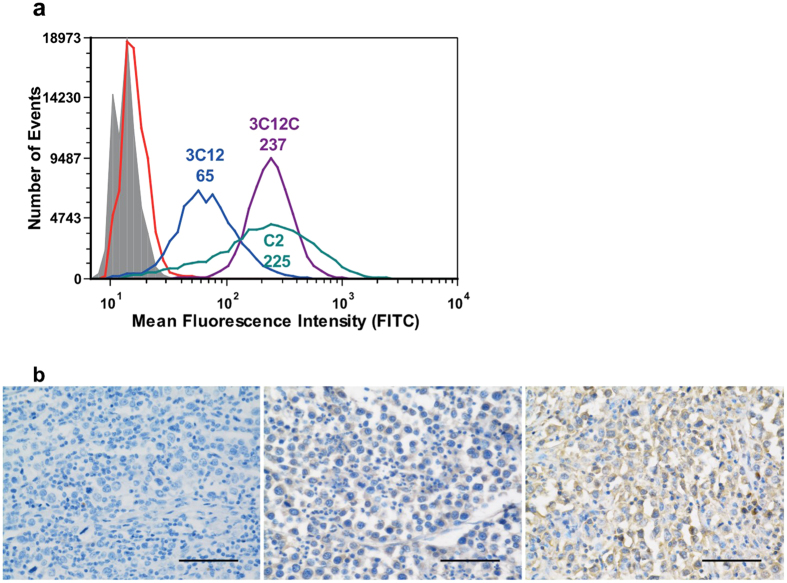
Testing of phage-derived, reformatted antibodies on native sources of their respective antigens. (**a**) Anti-CD83 mAbs were tested for binding to KM-H2 cells by flow cytometry. 3C12, isolated by biopanning against recombinant soluble CD83, is shown in blue. 3C12C is the affinity matured version of 3C12 (purple) and C2 was isolated by cell-based biopanning against CD83-GFP (teal). The control samples were cells only (shaded) and cells treated with secondary antibody only (red). The median fluorescence is indicated above the test samples. (**b**) Anti-CD117 mAbs were tested for binding to canine mast cell tumour tissue sections by immunohistochemistry. Sections were probed with secondary antibodies only (left), positive control anti-human c-Kit (middle) and A3 mAb isolated by cell-based biopanning on CD117-GFP (right). Scale bar represents 50 μm.

When biopanning on recombinant protein, the isolated clones must also be tested for binding to a native source of the antigen. The Hodgkin’s lymphoma cell line KM-H2, exhibits dendritic cell-like properties, including the expression of CD83^16^. Initial flow cytometry results using KM-H2 cells and phage particles showed no significant binding (including the positive control phage 3C12). This was attributed to a combination of low cell surface expression of CD83 and low avidity of the phage expressed scFv. As such, the phage scFv were reformatted to full length, bivalent IgG1 antibodies^15^. The reformatted C2 antibody was then compared with 3C12_IgG1^15^ (isolated by phage display against soluble CD83) and 3C12C_IgG1^17^ (light chain affinity matured version of 3C12) for binding to KM-H2 cells by flow cytometry (Fig. 6a). Results showed that all three antibodies bound to KM-H2 cells. As expected, the higher affinity 3C12C_IgG1 gave a greater fluorescence shift than 3C12_IgG1 (greater than three-fold). C2_IgG1 gave a broad peak with a median fluorescence similar to the 3C12C peak.

For canine CD117, the reformatted clone A3 was shown to bind to canine mast cell tumour tissue by immunohistochemistry (Fig. 6b). No native source testing has yet been performed for the CD11b specific clones due to a lack of material.

## Discussion

Therapeutic antibodies are often targeted towards membrane proteins where the purpose is to elicit an immune response against a target cell, deliver a conjugated drug to a target cell or block the receptor from binding to its natural ligand. Phage display generally uses purified, immobilized protein as the target for biopanning, but membrane proteins are difficult to purify as they contain hydrophobic domains, and may denature during the purification process. An option for screening antibody libraries for binders to membrane proteins is to use whole cells as the source of antigen, but the process of whole cell-based biopanning has its own limitations. Low abundance of the target protein amongst a high background of irrelevant antigens, and the inherent stickiness of phage particles makes this technique difficult and consequently relatively few reports of successful cell-based biopanning exist^18^,^19^,^20^.

A crucial step in the biopanning for antibodies against membrane proteins is to obtain a high surface density of the target antigen, as the low levels generally seen on native cells is insufficient to capture even high affinity antibodies from highly diverse libraries. Methods to display increased levels of antigen on cell surfaces by recombinant expression have been described in yeast^21^ and insect cells^22^, but mammalian cells offers the advantage of correct folding and post-translational processing of mammalian target proteins, even though their expression may be lower. The method described here uses transiently expressed membrane proteins in fusion with an intracellular GFP, in mammalian cells. This allows for the selection of cells expressing a high level of GFP that is directly proportional to the transfected receptor density.

The binding of the phage library is undertaken two days post-transfection and, due to the nature of transient transfection, the phage are exposed to cells with varying levels of antigen from none to high expression. Even after an initial depletion step on non-transfected cells, the non-expressing cells within the transfected population act to further sequester any binders against background antigens. After phage incubation and washing, the fused GFP is used to facilitate collection of only the high-expressing cells by FACS. This is similar to a method previously described where stable cell lines were stained and then mixed with a non-expressing cell line to sequester unwanted phage, prior to FACS^19^. However, that particular method requires the laborious and slow production of a stable cell line expressing the antigen of interest and subsequent cell staining, whereas our modification utilises transient transfection with a GFP fusion protein and can thus be performed in a much shorter time period. Transient transfection also allows the host cell line to be alternated between each round of biopanning, which changes the background antigens. We show that alternating the host cell line significantly increases the enrichment ratio of specific to non-specific binders, which is crucial to successful cell-based biopanning strategies.

It has previously been reported that biopanning on whole cells can result in the enrichment of phage clones which do not contain a scFv insert^23^. This occurs due to the co-elution of phage which are bound non-specifically to the cell surface, along with the specific phage bound through the scFv region. Any non-specific phage which either lack a scFv insert or have truncated or out-of-frame inserts, subsequently have a growth advantage during phage rescue. As such, we have incorporated the low pH wash recommended by Tur *et al.*^23^ to remove non-specifically bound phage prior to FACS. Additionally, the FACS process itself also helps to remove non-specifically bound phage due to the shearing force of flow cytometry.

Interestingly, we have observed that the affinity of the antibodies to CD83 and CD117 isolated by the described cell-based biopanning method are very strong for a naive library. For CD83, the isolated antibody showed better binding to KM-H2 cells than an antibody isolated from the same library but panned on soluble CD83 (Fig. 6). Furthermore, it bound as well as the affinity-matured version of the latter antibody. This is possibly because immobilisation of soluble antigen onto plastic surfaces results in a high density of antigen allowing for isolation of lower affinity binders, whereas cell-based biopanning forces higher affinity binders due to the lower antigen density. Preliminary studies with the antibodies to CD117 show affinity in the sub-nanomolar range; for example, reformatted antibody A3 binds to purified CD117 extracellular domain with an affinity constant (K_D_) of 9.25 × 10^−10^ M (see Supplementary Figure 1).

The successful isolation of antibodies using any cell-based biopanning method will depend on the accessibility of epitopes on the target protein. For the three antigens described here, we obtained the most number of unique binders for CD117. The extracellular domain of CD117 is relatively elongated with five immunoglobulin-like domains extending from the cell surface^24^, thereby exposing a greater number of epitopes than CD83 which has a single immunoglobulin-like domain sitting close to the cell surface^25^. The CD11b panning did not isolate as many binders during the monoclonal screening (Fig. 4), which was predicted from the polyclonal phage pool analysis (Fig. 2) as that showed less enrichment than for CD117. CD11b is normally associated as a heterodimer with CD18, and in the absence of an extrinsic ligand folds into a compact structure close to the cell membrane^26^. If it also forms this structure when expressed as a monomer, then it may also have few epitopes exposed. This is consistent with results reported by Hoogenboom *et al.*^20^ where binders were readily isolated against a membrane protein with a large and accessible extracellular domain, but the same panning strategy was unsuccessful with a more compact and highly glycosylated extracelluar domain^20^. We are further testing this method on a variety of different membrane protein structures. Multi-pass membrane proteins such as ion-channels contain even fewer exposed epitopes, and therefore it may be more difficult to isolate specific binders in these cases^20^.

Another potential limitation of the method as described is the possibility that the fused GFP molecule may interfere with the native structure of the extracellular domains of the membrane protein, which could occur if the protein spans the membrane multiple times or is a multi-subunit protein. This problem could be avoided by expressing the GFP from an internal ribosome entry site on the transfected transcript, resulting in proportional but separate translation. However, this will require case-by-case optimisation as our preliminary studies have shown that GFP expression is not always proportional under an IRES system and the level of target expression can be significant even in the absence of GFP expression^27^.

The cell-based, phage antibody library biopanning method described offers a protocol for the isolation of antibodies that bind membrane protein antigens in their native, membrane-bound state. This offers some advantages including removing the need for expression and production of purified, recombinant protein for biopanning campaigns. Importantly the methodology has incorporated steps to minimise enriching phage antibodies that bind irrelevant host cell antigens by rotating the host cell utilised for membrane protein expression, and by using rigorous, optimised washing steps to minimise non-specific binding. The method most likely works efficiently for membrane proteins that exhibit extended extracellular domains, with accessible antigenic determinants, rather than condensed protein structures that do not present many accessible regions. Accessibility may be hindered for other reasons such as extensive glycosylation of extracellular protein domains. Overall the method offers another strategy for the isolation of antibodies that bind membrane protein targets.

## Methods

### Protein Expression

The extracellular domain of human CD83 (6xHis tagged) and the phage-derived monoclonal antibody 3C12, reformatted to human IgG1-kappa were expressed and purified as described previously^15^. Additionally, the affinity matured 3C12C was prepared as described previously^17^.

The full-length sequence of human CD83 (NBCI Accession Q01151), bat CD11b (NCBI Accession ELK11001) and the extracellular and transmembrane domains of canine CD117 (Residues 1–544; NCBI Reference NP_001003181.1) were synthesized by Geneart (Germany). The cytoplasmic domain of CD117 was not included. The sequences included their native secretion sequences and were codon-optimised for expression in *Cricetulus griseus* (Chinese Hamster Ovary cells). The sequences were cloned in-frame with GFP into the mammalian expression vector pEGFP-N1 (Clontech), via NheI and BamHI restriction enzyme sites. DNA for transfection was prepared using PureLink HiPure Plasmid Maxiprep kit (Invitrogen).

CHO-S cells (Life Technologies) were cultured in CD-CHO with 8 mM Glutamax (Gibco) at 37 °C, 7.5% CO_2_ with shaking at 130 rpm. On the day of transfection, cells were prepared at ~3.0 × 10^6^ cells/mL at greater than 98% viability as measured using Cedex HiRes cell counter. For 10 mL culture volume, 20 μg of plasmid DNA was mixed with 1.25 mL OptiPro and incubated at room temperature for 30–60 sec; and concurrently 80 μL of PEI-Max was mixed with 1.25 mL OptiPro and incubated at room temperature for 30–60 sec. The PEI-Max complex was then added to the DNA complex by gentle pipetting, and incubated static at room temperature for 15 min. The DNA complex was then added to 10 mL culture in a shake-flask and mixed by gentle swirling. The culture was incubated at 37 °C, 7.5% CO_2_ with shaking at 130 rpm for 4 hrs, after which the culture was diluted 1:2 (v:v) with CD-CHO and anti-clumping agent added to 0.4% (v/v). The culture was incubated at 32 °C, 7.5% CO_2_, 130 rpm for up to 6 days.

Suspension HEK-293 cells (Life Technologies) were transfected in a similar manner, but were grown in Freestyle-293 media and transfected at a concentration of ~2.2 × 10^6^ cells/mL.

### Flow Cytometry Analysis of CD83 expression

Approximately 1 × 10^6^ cells were collected each day post-transfection for analysis of CD83-GFP expression by flow cytometry. The cells were washed twice with PBS-2% (v/v) FCS, then resuspended to 2 × 10^6^ cells per mL in PBS-2% FCS. Aliquots of 2 × 10^5^ cells were incubated either alone or with 10 μg/mL final concentration of 3C12 antibody for 1 hr at room temperature, followed by two washes with PBS-2% FCS. The cells were then incubated with secondary antibody, Goat Anti-human IgG Fc component-APC conjugate (Jackson Immunoresearch) for 30 min at room temperature. Cells were washed twice as above, then analysed for GFP and APC on the Accuri C6 flow cytometer (BD Biosciences).

Similarly, KM-H2 cells^16^ cultured in RPMI with Glutamax and 10% FCS at 37 °C, 5% CO_2_ were used to test antibody binding to a native source of CD83 by flow cytometry, but were analysed using a Partec Cube8 flow cytometer (Partec, Germany). The secondary antibody was FITC-labelled anti-human IgG (Southern Biotechnologies).

### Biopanning

For the biopanning of CD83-GFP, the ‘Sheets’ human naive library^28^, with a reported diversity of 6.7 × 10^9^ was used, kindly supplied by Prof. James Marks (University of California San Francisco). For the biopanning of CD117-GFP and CD11b-GFP, the newly generated mAbLAb library prepared as described in the Supplementary Protocol 1, using the primers listed in Supplementary Table 1, was used. The diversity of this library (5 × 10^9^) is similar to the Sheets library, but while the Sheets library consists mainly of scFv with Vh3 heavy chains, the mAbLAb library contains excellent representation of all Vh families (see Supplementary Figure 1).

For the biopanning of CD83-GFP, the first three rounds were performed on transfected CHO-S cells followed by one round on transfected HEK cells. For the biopanning of CD117-GFP and CD11b-GFP, Round 1 was performed on CHO-S, Round 2 on HEK, Round 3 on CHO-S and Round 4 on HEK transfected cells. All biopanning rounds were performed on cells collected two days post-transfection.

The biopanning procedure is shown diagrammatically in Fig. 1. Approximately 1 × 10^7^ non-transfected cells were collected, washed in PBS then blocked in 5 mL 2% (w/v) milk-PBS (MPBS), rotating for 30 min at 4 °C. Concurrently, ~10^12^ phage particles from the rescued library stock were also blocked in MPBS. The blocked phage were then added to the blocked cells and incubated rotating for 1 hr at 4 °C. The phage/cell suspension was centrifuged at 500 g for 3 min, then the supernatant containing non-bound phage was used to resuspend washed transfected cells, then incubated at 4 °C for 1 hr. The cells were then washed three times using pH 5 Wash Buffer (PBS with the pH lowered to 5.0 using citric acid, and containing 0.1% (v/v) Tween-20), followed by 3 washes in PBS, pH 7.4. The washed cells were sorted using a BD Influx^TM^ cell sorter at 27 PSI, 100 micron nozzle, 200 mW 488 nm laser exciting GFP (528/38BP collection filter). After gating to exclude cellular debris, based on Forward and Side scattered light parameters, and excluding doublets, single cells with high GFP fluorescence (top 10%) were collected into PBS. The sorted cells were pelleted by centrifugation at 800 g for 5 min, then the phage eluted by incubation in 0.5 mL of 75 mM Citrate, pH2.3 for 6 min at room temperature. After centrifugation at 800 g for 5 min, the supernatant was neutralized with 0.5 mL 1M Tris, pH 7.5. The phage eluate was used to infect 10 mL XL1-Blue cells at OD_600_ 0.6–0.8.

Phage particles were prepared for subsequent rounds of biopanning, by amplification and rescue using M13K07 helper phage as per standard procedures^12^. The Round 1 amplified phage was used as the phage input for Round 2 biopanning, and so forth.

### Polyclonal Phage ELISA

Pools of purified phage from the library stock (Sheets), and the Round 1, Round 2, Round 3 and Round 4 amplified stocks from the CD83-GFP biopanning were tested for binding to soluble CD83 using ELISA. Nunc Maxisorp 96-well plates were coated with 5 μg/mL CD83 in PBS overnight at room temperature. The wells were washed three times with PBS and then blocked for 1 hr with MPBS. Phage pools (~10^11^ phage particles) were also blocked in MPBS. Dilutions of the blocked phage were then added to the CD83 coated plate and incubated for 1 hr at room temperature. The wells were washed three times with PBS containing 0.1% (v/v) Tween-20 (PBST), and then 200 μL secondary antibody added (1/5000 dilution of HRP/Anti-M13 Monoclonal Conjugate-GE Healthcare) and incubated for 1 hr at room temperature. The wells were washed three times with PBST, followed by addition of 100 μL of 3,3′,5,5′-Tetramethylbenzidine (TMB) Liquid Substrate (Sigma-Aldrich). The reaction was stopped after 10 mins using 100 μL 1 M sulphuric acid. Absorbance was measured at 450 nm using BioTek Powerwave HT Microplate Reader (Millenium Science).

### Monoclonal Phage ELISA

Individual phage clones from either the third or fourth biopanning round on CD83-GFP were tested for binding to soluble CD83 by ELISA. Glycerol stock of XL1-Blue containing eluted phage from Rounds 3 and 4 were streaked onto 2YT-AmpGlu media to obtain individual colonies. Colonies were grown in 96-well plates in 2YT-AmpGlu followed by phage production using M13K07 helper phage rescue. Phage were produced overnight in 2YT-AmpKan, then the culture supernatant used for ELISA. ELISA plates were coated with 3 μg/mL soluble CD83 in PBS overnight at room temperature. Phage rescue supernatant and the coated plate were blocked in MPBS for 1 hr at room temperature. Blocked phage was then transferred to the ELISA plate, and the ELISA was continued as described above.

### Flow Cytometry Analysis of Phage Pools and Clones

For each of the CD83-GFP, CD117-GFP and CD11b-GFP biopannings, pools of purified phage from the respective library stock, and the Round1, Round2 and Round3 amplified stocks were tested for binding to transfected cells using flow cytometry. CHO cells were transfected as described above with either the CD83-GFP, CD117-GFP or CD11b-GFP expression plasmids, and used two days post-transfection. ~2 × 10^5^ cells were washed twice in 5 mL PBS, then blocked in 5 mL MPBS. Phage pools (~10^11^ phage particles) were also blocked in MPBS. Cells were then incubated either alone or with blocked phage from the various pools for 1 hr at 4 °C. The cells were washed twice with PBS, then incubated with 1/200 diluted Anti-M13 Monoclonal Antibody (GE Healthcare), conjugated to Dylight 594 (Thermo Scientific), for 30 min at room temperature. The cells were washed twice in PBS, then analysed for GFP and DL594 fluorescence on a Partec Cube8 flow cytometer. Similarly, flow cytometry was also used to analyse individual phage clones, prepared as described above for monoclonal phage ELISA.

### Sequencing of Clones

Phagemid DNA was isolated from individual clones which showed specificity for their target antigen. Clones were grown overnight in 2YT-AmpGlu media, then phagemids isolated using Purelink Hipure Plasmid Mini Prep kit (Invitrogen). Phagemid scFv inserts were sequenced by Sanger sequencing at the Australian Genome Research Facility (AGRF, Brisbane Australia), using phagemid-derived forward and reverse primers.

### Reformatting and Expression of Phage Clones

Phage clones of interest were reformatted to whole human IgG1 using ligation-free cloning into mammalian expression vectors, expressed in CHO cells and purified by Protein A affinity chromatography as described previously^15^.

### Immunohistochemistry

Paraffin embedded canine mast cell tumour sections were prepared and supplied by Dr Rachel Allavena (The University of Queensland Veterinary School, Gatton). The sections were dewaxed in xylene, rehydrated in graded alcohols and deionized in distilled water. Heat-induced antigen retrieval was conducted by immersing the slides in 0.01 M Citric Acid-Based Buffer, pH 6.0 (Vector) for three periods of 5 mins in a microwave (650W). After cooling at room temperature (RT) for 25 mins, the endogenous peroxidase activity was quenched by incubation with 1% (w/v) hydrogen peroxide in methanol for 40 mins at RT in the dark. The slides were washed in TBS-T then blocked in 5% (v/v) goat serum (Abcam) in TBS-T for 30 min at RT. The slides were then treated with Endogenous Avidin/Biotin Blocking Kit (Abcam) as per the manufacturer’s instructions. The reformatted phage clone A3, and a commercial positive control mouse anti-human CD117 (BD Biosciences) shown to cross-react with canine CD117^29^, were diluted to 30 μg/mL in TBS, pH 7.4, and incubated on the slides overnight at 4 °C in a covered humidity chamber. A negative control slide was incubated concurrently in TBS buffer. The slides were then washed in TBS-T, followed by incubation with biotinylated Anti-human Fc (Life Technologies) or Anti-mouse IgG (Life Technologies), diluted 1/500 in TBS for 30 min at RT. After washing in TBS-T, the slides were incubated with ABC substrate solution (Vector) for 30 mins, then developed with DAB for 8 mins followed by distilled water rinsing. Tissue sections were counterstained with hematoxylin for 45 sec. The slides were then dehydrated, fixed and mounted with xylene mounting medium.

## Additional Information

**How to cite this article**: Jones, M. L. *et al.* Targeting membrane proteins for antibody discovery using phage display. *Sci. Rep.* **6**, 26240; doi: 10.1038/srep26240 (2016).

## Supplementary Material

Supplementary Information
