# Supplementary material for: Targeting membrane proteins for antibody discovery using phage display
Source: Sci Rep. 2016 May 18;6:26240. doi: 10.1038/srep26240 (PMC4870581; doi:10.1038/srep26240)
Supplement: Supplementary Information [file srep26240-s1.pdf]

Supplementary Material

Manuscript Title:

Targeting membrane proteins for antibody discovery using phage display

Authors:

Martina L Jones[1,*], Mohamed A Alfaleh[1,2], Sumukh Kumble[1], Shuo Zhang[1], Geoffrey W Osborne[1,3], Michael Yeh[1], Neetika Arora[1], Jeff Jia Cheng Hou[1], Christopher B Howard[1], David Y Chin[1], Stephen M Mahler[1]

[1] Australian Institute for Bioengineering and Nanotechnology, The University of Queensland, St Lucia Queensland 4072 Australia

[2] School of Pharmacy; King Abdulaziz University, 21589 Jeddah, Kingdom of Saudi Arabia

[3] Queensland Brain Institute, The University of Queensland, St Lucia Queensland 4072 Australia

* Correspondence: martina.jones@uq.edu.au

Supplementary Methods

mAbLAb Library Generation

Phage Display vector: A DNA fragment was synthesised by Geneart (Germany), consisting of (from 5' to 3'): a BssHII restriction site, ribosome binding site, the pelB leader sequence, SfiI, XhoI, NotI restriction sites, 6xHis tag, myc tag, amber codon, M13 phage g3p sequence, stop codon and BsmFI restriction site. The coding region was codon-optimised for expression in *E.coli*. This fragment was cloned into the backbone phagemid vector pBluescript II SK(+) (Agilent Technologies) using BssHII and BsmFI, retaining the vector-derived Lac promoter but removing the multiple cloning site and LacZ gene. The resulting phage display vector was named pPhNBF.

Based on pPhNBF cloning sites, degenerate primers were designed to cover the entire repertoire of human heavy, kappa and lambda chain variable regions using sequences reported in the IMGT database (IMGT-repertoire, Allele Align). Primers were ordered from Sigma (Supplementary Table 1).

Library Construction

The use of donated human blood samples for the generation of an antibody library was approved by The University of Queensland Medical Research Ethics Committee (Project number 2012000842). Buffy coat samples were obtained from the Australian Red Cross Blood Service from existing healthy blood donors. No information regarding donor age, gender or ethnicity was disclosed. cDNA library was generated using oligo-dt from total RNA isolated from buffy coat samples. cDNA was used as template in first-round PCRs.

For the heavy chain variable sequences, the first-round PCRs utilised nine forward primers with either IgM_Rev or IgG_Rev separately to amplify both naive and affinity-matured variable regions, respectively. The PCR products were pooled, purified and then used as template in second-round PCRs with forward primer (SfiI_For) pair with each of the three J-region reverse primers. Part of the $(G_4S)_3$ peptide linker was included in J-region reverse primers for subsequent overlap PCRs.
Kappa chain variable sequences were amplified by PCR using six forward primers with a single J-region reverse primer. Similarly for the lambda chain variable region, six forward primers were used with two J-region reverse primers. Part of the $(G_4S)_3$ peptide linker was included the forward primers for subsequent overlap PCRs.

Purified heavy and light chain regions were then spliced together into an scFv format using splice overlap extension of the $(G_4S)_3$ peptide linker region, and amplified with SfiI_For and NotI_Rev primers. The final scFv product was purified and restriction digested with SfiI and NotI for ligation with pPhNBF. The ligated products were transformed intoTG1 electrocompetent cell (Lucigen) by electroporation. Transformants were plated out on YT agar plates, supplemented with 2% (w/v) glucose and 100 µg/mL ampicillin. The resulting colonies were collected and stored in aliquots of glycerol stocks, from each transformation, at -80°C. Prior to panning, phage particles were rescued from the library and aliquoted and stored in PBS-20% (v/v) glycerol at -80°C.

Random colonies from transformation plates were picked, and the scFv insert amplified by PCR and sequenced by Sanger Sequencing (AGRF, Brisbane Australia). The sequences were then analysed using IMGT-V-Quest to determine variable gene families.

Affinity analysis of antibody binding to Canine CD117 extracellular domain

The extracellular domain (residues 1-523) of canine CD117 (NCBI Reference NP_001003181.1) was PCR amplified from the GFP fusion sequence described earlier, and ligated to a human immunoglobulin Fc domain sequence previously cloned into pcDNA3.1(+) (Life Technologies). The plasmid (600µg) was mixed with 30mL OptiPro media (Life Technologies), then mixed with 2.4mL PEI-Max (Polysciences) diluted in 30mL OptiPro media and incubated at room temperature for 15min. The DNA complex was then added to 100mL CHO-S cells at $3.0x10^6$ cells/mL in CD-CHO with 8mM Glutamax (Gibco), then the cells were incubated at 37°C, 7.5% CO_2, 130rpm for 4h. The culture was then diluted 1:1 (v:v) with CD-CHO, 8mM Glutamax, 0.4% anti-clumping agent, and incubated at 32°C, 7.5% CO_2, 130rpm for 10 days. The CD117ecd-hFc fusion protein was purified from the culture supernatant using Protein A affinity chromatography on a mAbSelect SuRE column (GE Healthcare), as per the manufacturer's instructions. The purified protein was confirmed by ELISA to bind to Mouse Anti-human CD117 (BD Biosciences) which is known to cross-react with canine CD117 (data not shown).

The interaction kinetics of the reformatted antibody clone A3, isolated by panning on CD117-GFP expressing cells, and CD117ecd-hFc fusion protein was analysed by surface plasmon resonance on a Biacore T200 instrument (GE Healthcare). Purified CD117ecd-hFc protein was diluted in 10mM sodium acetate buffer, pH 5 to 10 µg/mL and immobilised onto flowcell 4 of a CM5 chip to 100 RU using an Amine Coupling Kit (GE Healthcare) as per the manufacturer's instructions. Single cycle kinetics was used to sequentially inject 0.34, 1.02, 3.06, 9.18 and 27.54 nM of purified reformatted

A3 antibody over the immobilised surface at 100 µL/min for 180 sec, with a final dissociation phase of 600 sec. The binding surface was regenerated using 10mM glycine, pH 2.0 for 30 sec at 10 µL/min. Flow cell 3, containing no immobilised CD117ecd-hFc, was used for reference subtraction along with a buffer-only cycle. The sensorgram was analyzed using BiaEvaluation software (GE Healthcare) and a monovalent Langmuir (1:1) binding model was used to fit kinetic variables.

Supplementary Table 1: Primers used in the construction of the mAbLAb human naive scFv library

Oligo Name	Sequence 5' to 3'
HEAVY CHAIN PRIMERS	
VhA_For **(Vh1,7)**	atttaaggcccagccggccatggccCAGGTBCAGCTKGTRCAGTCTGG
VhB_For **(Vh1)**	atttaaggcccagccggccatggccCARATGCAGCTGGTGCAGTCTGG
VhC_For **(Vh2)**	atttaaggcccagccggccatggccCAGRTCACCTTGARGGAGTCTGG
VhD_For **(Vh3)**	atttaaggcccagccggccatggccSAGGTGCAGCTGKTGGAGTCTG
VhE_For **(Vh3)**	atttaaggcccagccggccatggccGAGGTGCAGCTGGTGGAGTCTCG
VhF_For **(Vh4)**	atttaaggcccagccggccatggccCAGSTGCAGCTGCAGGAGTCGGG
VhG_For **(Vh4)**	atttaaggcccagccggccatggccCAGGTGCAGCTACARCAGTGGGG
VhH_For **(Vh5)**	atttaaggcccagccggccatggccGARGTGCAGCTGGTGCAGTCTGG
VhI_For **(Vh6)**	atttaaggcccagccggccatggccCAGGTACAGCTGCAGCAGTCAGG
IgG_Rev	GATGGGCCCTTGGTGGARGC
IgM_Rev	GGTTGGGGCGGATGCACTCCC
SfiI_For	atttaaggcccagccggccatggcc
JhA_Rev	*AGAGCCACCTCCGCCTGAACCGCCTCCACC*TGAGGAGACGGTGACCAGGGTBCC
JhB_Rev	*AGAGCCACCTCCGCCTGAACCGCCTCCACC*TGAAGAGACGGTGACCATTGTCCC
JhC_Rev	*AGAGCCACCTCCGCCTGAACCGCCTCCACC*TGAGGAGACGGTGACCGTGGTCCC
KAPPA PRIMERS	
VkA_For **(Vk1)**	*ggcggaggtggctctggcggtggcgctagc*GACATCCAGWTGACCCAGTCTCC
VkB_For **(Vk2,3,4,6)**	*ggcggaggtggctctggcggtggcgctagc*GAWATTGTRATGACNCAGTCTCC
VkC_For **(Vk3,6)**	*ggcggaggtggctctggcggtggcgctagc*GAAATTGTGYTGACDCAGTCTCC
VkD_For **(Vk1)**	*ggcggaggtggctctggcggtggcgctagc*GCCATCCGGATGACCCAGTCTCC
VkE_For **(Vk5)**	*ggcggaggtggctctggcggtggcgctagc*GAAACGACACTCACGCAGTCTCC
VkF_For **(Vk1)**	*ggcggaggtggctctggcggtggcgctagc*GTCATCTGGATGACCCAGTCTCC
Jk_Rev_NotI	atgatgtgcggccgcacgTTTRATHTCCASYTTKGTCCC
Linker_For	*ggcggaggtggctctggcggtgg*
LAMBDA PRIMERS	
VLA_For **(VL1)**	*ggcggaggtggctctggcggtggcgctagc*CAGTCTGTGYTGACKCAGCC
VLB_For **(VL2)**	*ggcggaggtggctctggcggtggcgctagc*CAGTCTGCCCTGACTCAGCC
VLC_For **(VL3)**	*ggcggaggtggctctggcggtggcgctagc*TCCTATGAGCTGACDCAG
VLD_For **(VL4,5,9,11)**	*ggcggaggtggctctggcggtggcgctagc*CAGCCTGTGCTGACTCAGCC
VLE_For **(VL4)**	*ggcggaggtggctctggcggtggcgctagc*CAGCYTGTGCTGACTCAATC
VLF_For **(VL6,7,8,10)**	*ggcggaggtggctctggcggtggcgctagc*CAGRCTGTGSTGACTCAG

JLA_Rev	atgatgtgcggccgcaccTAGGACGGTCASCTTGGTCCC
JLB_Rev	atgatgtgcggccgcaccKAGGACGGTCAGCTGGGTBCC
NotI_Rev	atgatgtgcggccgcac

Upper case letters represent homology to human immunoglobulin sequences

Underlined letters correspond to restriction enzyme sites

Italic letters correspond to the peptide linker

Bold represents the variable region family to which the primer will anneal.

Degenerate codes: R: A/G; Y: C/T; S: G/C, W: A/T; K: G/T; B: C/G/T; D: A/G/T; H: A/C/T; N: A/C/G/T

Supplementary Results

mAbLAb Library Analysis

The final library size was determined to be 5×10^9 transformants. Sequence analysis of 48 random clones from the library showed that 68% of clones contain full-length, in-frame scFv. From these full-length clones, there is approximate equal representation of different variable region families, and kappa and lambda chains (Supplementary Figure 2). Unlike other reported libraries where the diversity reflects the *in vivo* diversity[1], the mAbLAb library was designed to increase representation of variable region families that are normally under-represented *in vivo*, by amplifying each family separately and then normalising the amount of amplified DNA used in subsequent steps. This may facilitate an overall increase in combinatorial diversity by pairing of rarer chains. From a limited number of antigen-selected clones, we have isolated scFv from the less common families Vh1 and Vh6, whereas selected antibodies from other libraries are mostly Vh3.[1-5] The length of Vh-CDR3 in the unselected library ranged from 6 to 30 residues (average 12.8), and in selected clones from 10 to 20 residues.

Affinity analysis of antibody binding to Canine CD117 extracellular domain

Surface plasmon resonance analysis was used to investigate the binding kinetics of the interaction between reformatted antibody A3, isolated by panning on CD117-GFP expressing cells, and purified CD117ecd-hFc fusion protein. The blank-subtracted single cycle kinetics sensorgram is shown in Supplementary Figure 2. The associate rate (k_a) was 8.09×10^5 $M^{-1}s^{-1}$, the dissociation rate (k_d) was 7.49×10^{-4} s^{-1}, and the calculated affinity constant (K_D) was 9.25×10^{-10} M, with a Chi^2 value of 0.382.

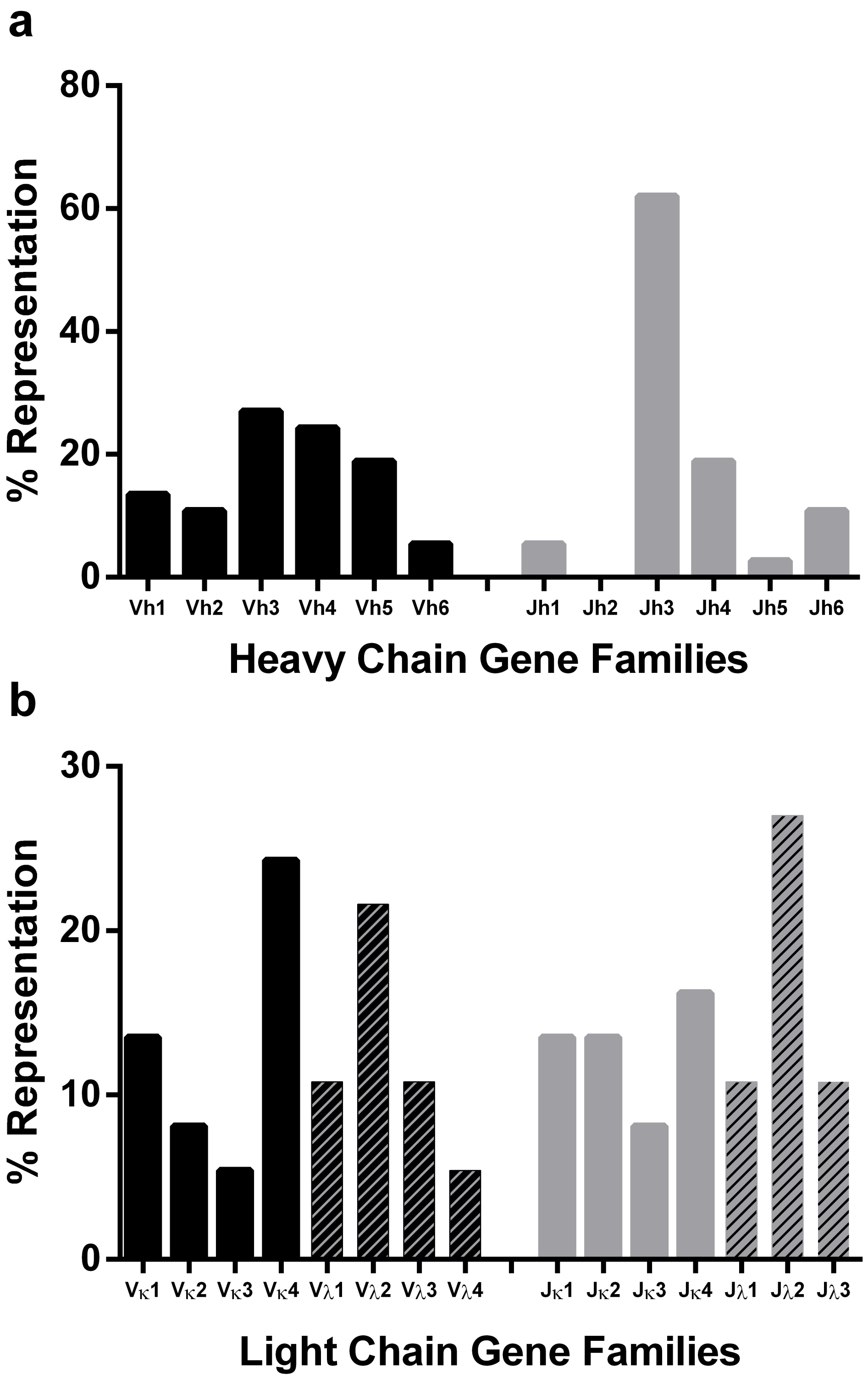

Supplementary Figure 1: Antibody gene family representation present in the mAbLAb library, from 37 full-length scFv clones randomly selected from the library. (A) Representation of heavy chain gene families, for the variable and J-regions. (B) Representation of kappa (κ) and lambda (λ) light chain gene families, for the variable and J-regions.

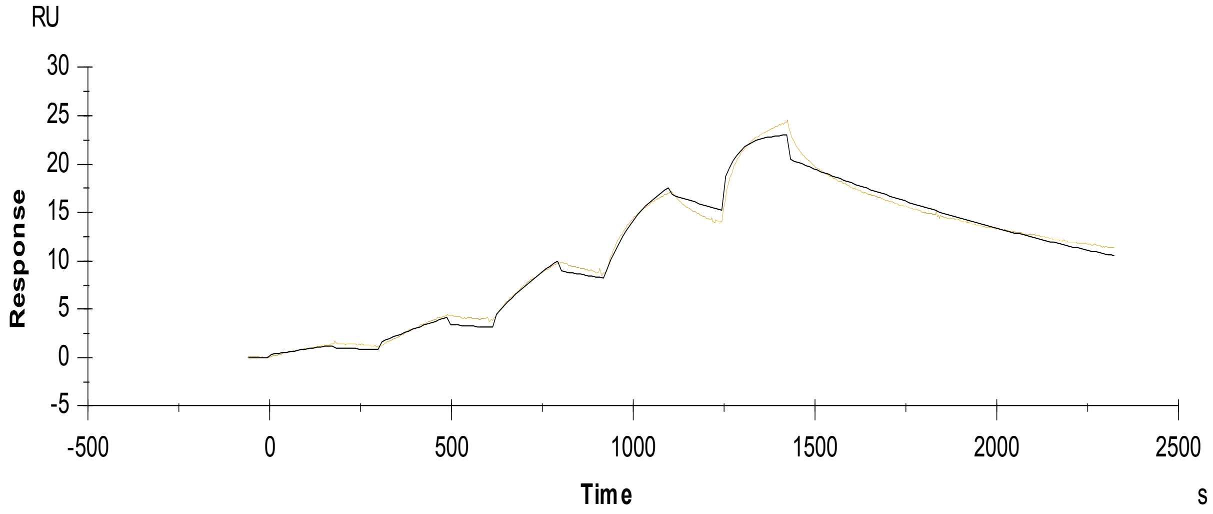

Supplementary Figure 2: Surface Plasmon Resonance single-cycle kinetics analysis of reformatted antibody A3 against CD117ecd-hFc fusion protein. Reformatted antibody A3 (isolated by panning on cells expressing CD117-GFP) was sequentially injected at increasing concentrations over a CM5 chip with immobilised CD117ecd-hFc fusion protein. The plot shows the blank-subtracted sensorgram (yellow line) and the 1:1 binding model curve fit (black line) generated by the BiaEvaluation software.

1. Kugler, J. et al. Generation and analysis of the improved human HAL9/10 antibody phage display libraries. *BMC Biotechnol* **15**, 10 (2015).
2. Sheets, M.D. et al. Efficient construction of a large nonimmune phage antibody library: the production of high-affinity human single-chain antibodies to protein antigens. *Proc Natl Acad Sci U S A* **95**, 6157-6162 (1998).
3. Griffiths, A.D. et al. Isolation of high affinity human antibodies directly from large synthetic repertoires. *EMBO J* **13**, 3245-3260 (1994).
4. Vaughan, T.J. et al. Human antibodies with sub-nanomolar affinities isolated from a large non-immunized phage display library. *Nat Biotechnol* **14**, 309-314 (1996).
5. Nissim, A. et al. Antibody fragments from a 'single pot' phage display library as immunochemical reagents. *EMBO J* **13**, 692-698 (1994).
